# Supplementary material for: The mitotic chromosome periphery modulates chromosome mechanics
Source: Nat Commun. 2025 Jul 10;16:6399. doi: 10.1038/s41467-025-61755-5 (PMC12246412; doi:10.1038/s41467-025-61755-5)
Supplement: Supplementary file 1 — Supplementary Information [file 41467_2025_61755_MOESM1_ESM.pdf]

Supplementary Figures: The Mitotic Chromosome Periphery Modulates Chromosome Mechanics

Tania Mendonca[1,2@], Roman Urban[3], Kellie Lucken[1], George Coney[1], Neil M. Kad[3], Manlio Tassieri[4@], Amanda J. Wright[2@] and Daniel G. Booth[1@]

[1]Biodiscovery Institute, School of Medicine, University of Nottingham, Nottingham NG7 2RD, UK
[2]Optics and Photonics Research Group, Faculty of Engineering, University of Nottingham, Nottingham NG7 2RD, UK
[3]School of Biosciences, University of Kent, Canterbury CT2 7NH, UK
[4]Division of Biomedical Engineering, James Watt School of Engineering, Advanced Research Centre, University of Glasgow, Glasgow G11 6EW, UK

[@]Corresponding authors:
Dr Daniel G. Booth daniel.booth@nottingham.ac.uk,
Dr Tania Mendonca tania.mendonca@nottingham.ac.uk,
Prof Amanda J. Wright amanda.wright@nottingham.ac.uk,
Dr Manlio Tassieri manlio.tassieri@glasgow.ac.uk

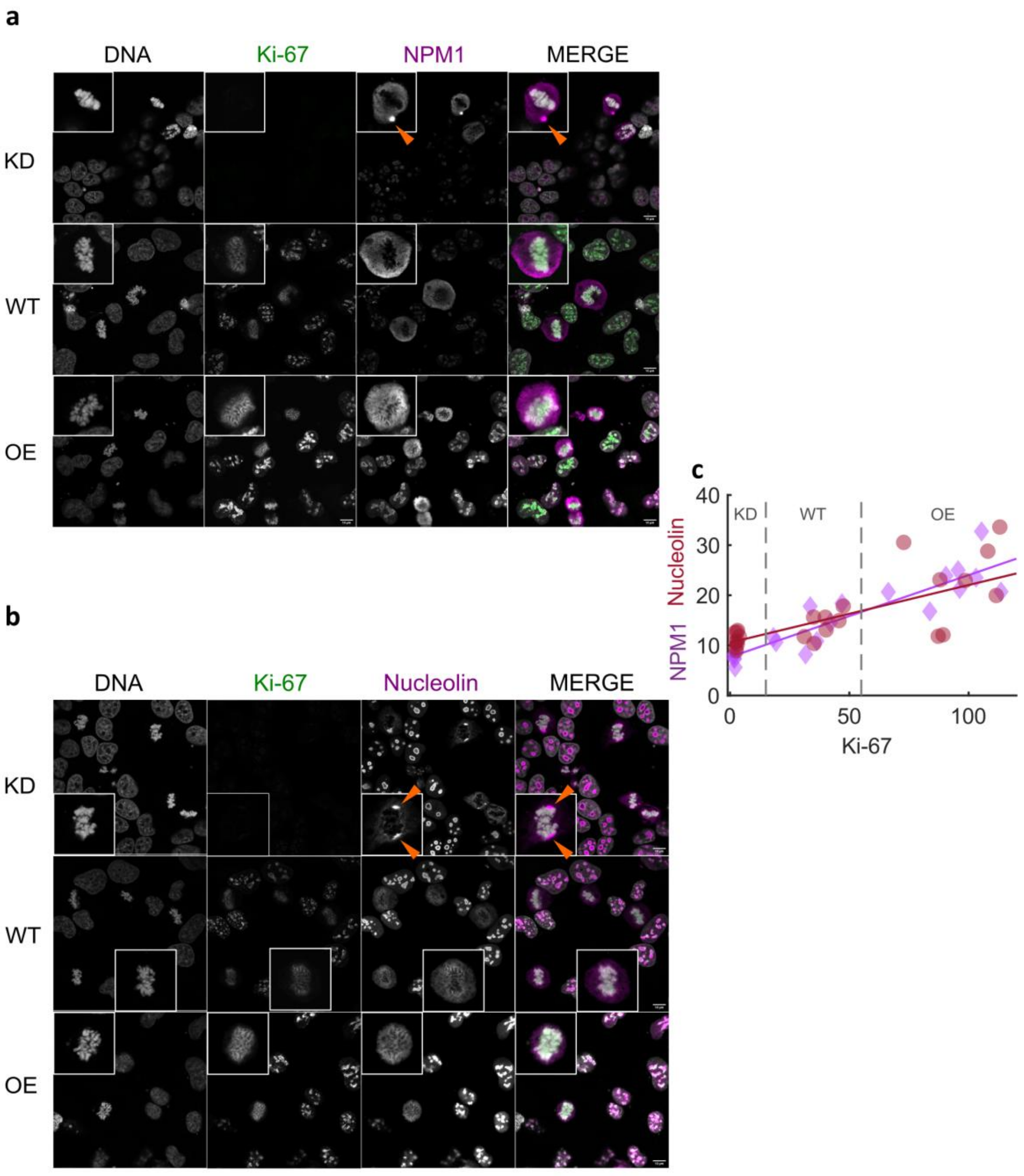

Fig. S1. Ki-67 levels dictate localisation of the periphery proteins, nucleophosmin (NPM1) and nucleolin at mitotic chromosomes. Confocal images of Ki-67-mEmerald cells with wild type (WT), knockdown (KD) or overexpressed (OE) levels of Ki-67, immuno-labelled for a. NPM1 and b. nucleolin. Cells were counterstained for DNA with DAPI. NPM1 and nucleolin aggregates (orange arrows) in Ki-67 KD mitotic cells. Insets show zoomed in individual mitotic cells from images. Scale bar = 10 μm. c. Mean fluorescence intensity (arbitrary units) of NPM1 and nucleolin at the chromosome periphery vs intensity of Ki-67 in early mitotic WT n = 7 each, KD n = 9 (Nucleolin) and n = 6 (NPM1) and OE n = 10 (Nucleolin) and n = 8 (NPM1) Ki-67-mEmerald cells with linear regression fits (NPM1 vs Ki-67: $r2 = 0.8$, $p < 0.0001$ and nucleolin vs Ki-67: $r2 = 0.6$, $p < 0.0001$)

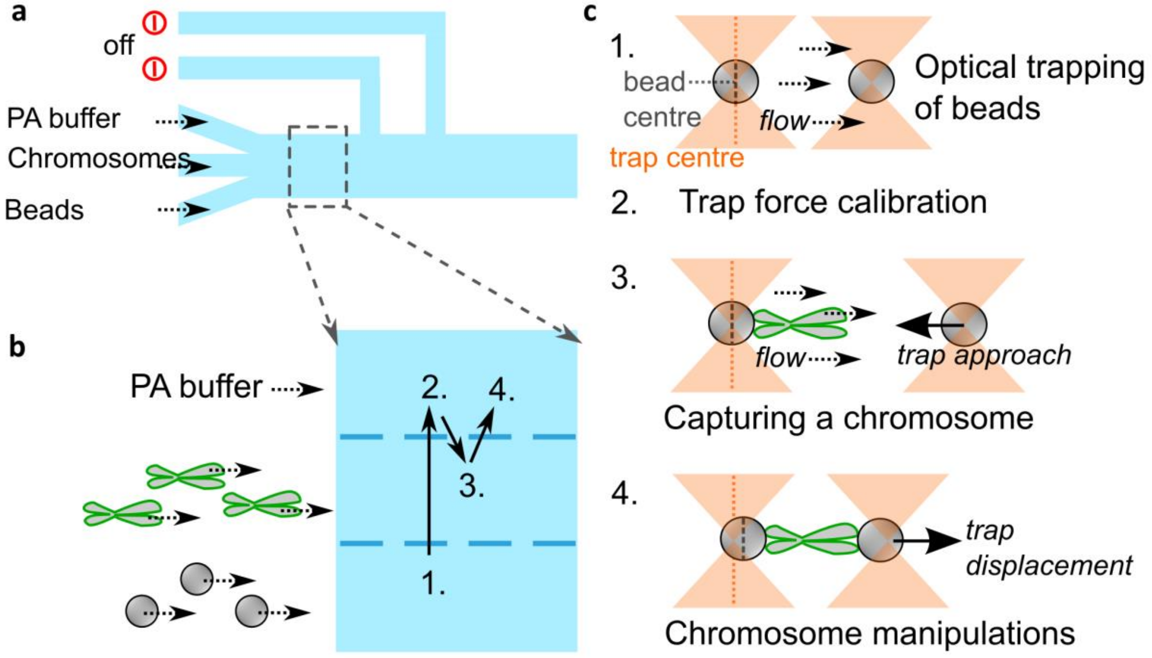

Fig. S2. Assembling the chromosome dumbbell unit for single-chromosome manipulations a. Schematic of the u-Flux microfluidics flow cell (type c1). Only three channels were used. b. Sub-region of the flow cell mapping the positions of each step in dumbbell assembly procedure. c. The four main steps to all chromosome manipulation experiments: 1. Optical trapping of beads as they flow through channel 1. Dotted vertical lines represent centres of the optical trap (orange) and bead (grey), only shown on one side but applies to both. Dotted arrows represent direction of fluid flow. 2. Each new pair of trapped beads were calibrated to accurately measure trapping force on both beads. Fluid flow is switched off during calibration. 3. A single chromosome is captured by binding to one bead first and then bringing the second bead into contact to form the dumbbell. 4. Single chromosomes are stretched in the absence of fluid flow by displacing the position of one optical trap while leaving the second stationary. The stretched chromosome displaces the bead handles such that the bead centres and optical trap centres are no longer coincident. Schematics shown are not to scale.

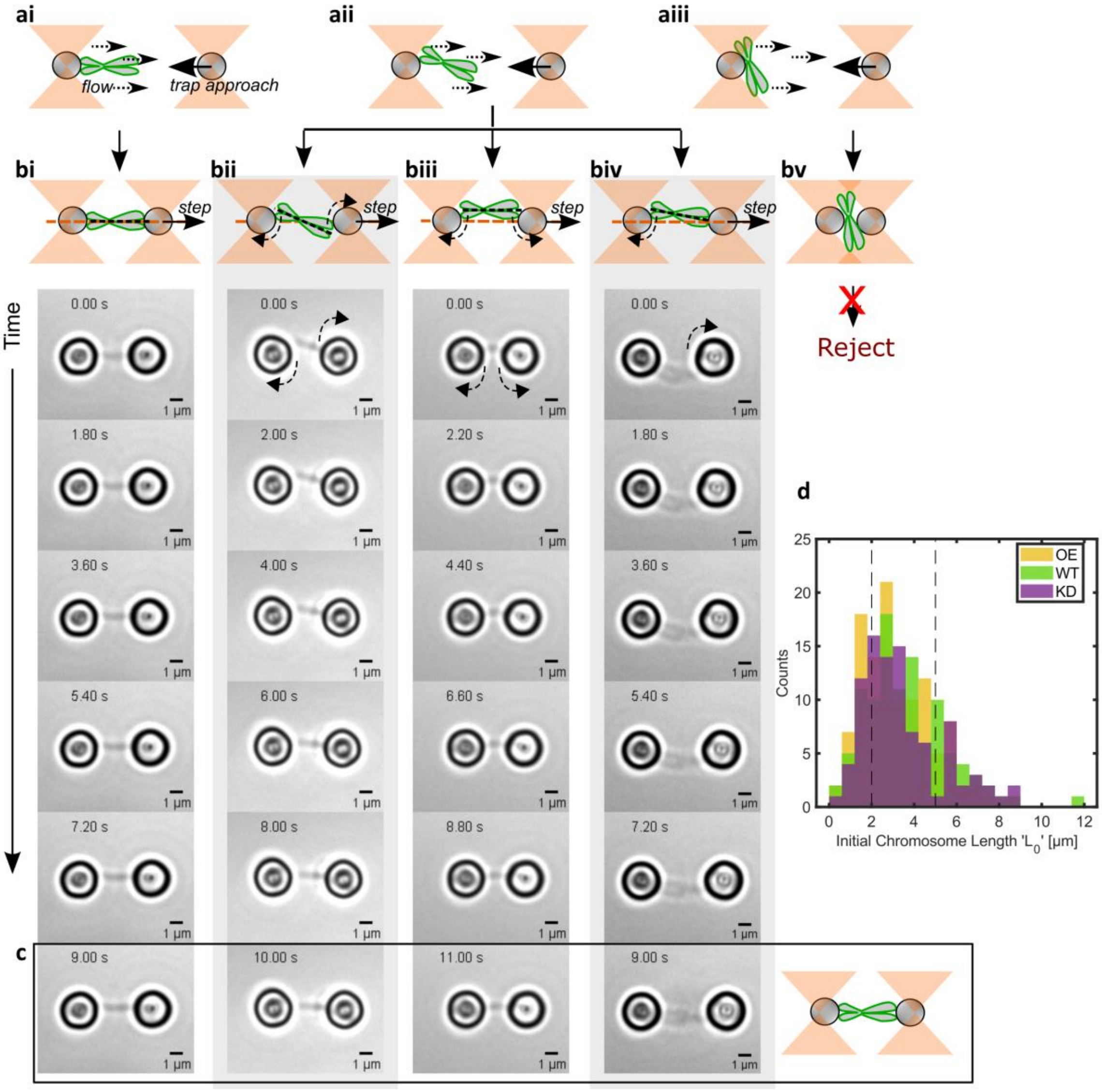

Fig. S3. Chromosome capture configurations Schematic of capture configurations; ai. telomeric capture at first bead of two sister chromatids or aii. one chromatid, and aiii. non-telomeric capture. bi-v. Possible dumbbell conformations with bi. ideal attachment of both sister chromatids at both beads, bii. only one but different chromatid at each bead biii. only one but the same chromatid at both beads and biv. one chromatid at one bead and two at the second bead. A short step was applied to one optical trap which rotated one or both beads (dashed curved arrows) such that the long axis of the chromosome (black dashed line) co-aligns with the X-axis of the dumbbell (orange dashed line) in cases bii-iv and brings the dumbbell into the desired position. Video montages are shown below each case to illustrate time series of dumbbell self-correction. bv. Non-telomeric captures are discarded c. Final dumbbell conformation before experimental manipulations. Schematics shown are not to scale. d. Histogram of natural lengths 'L_0' of captured chromosomes with size exclusion limits represented by black dashed lines.

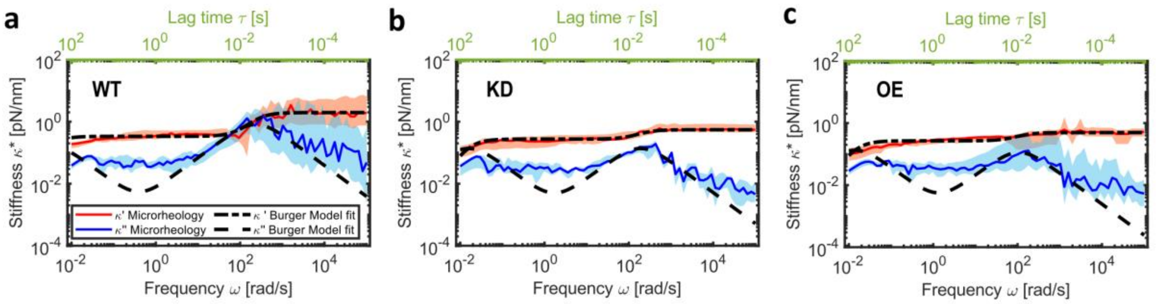

Fig. S4. Burgers Model fits (Fits to κ' depicted as dash-dotted lines and fits to κ'' as dashed lines) overlaid on broadband microrheology data (κ' in red and κ'' in blue, median and 95% CI) of a. Wild-type (WT) n = 14 chromosomes. b. Ki-67 knockdown (KD) n = 16 chromosomes and c. Ki-67 over-expressed (OE) n = 15 chromosomes.

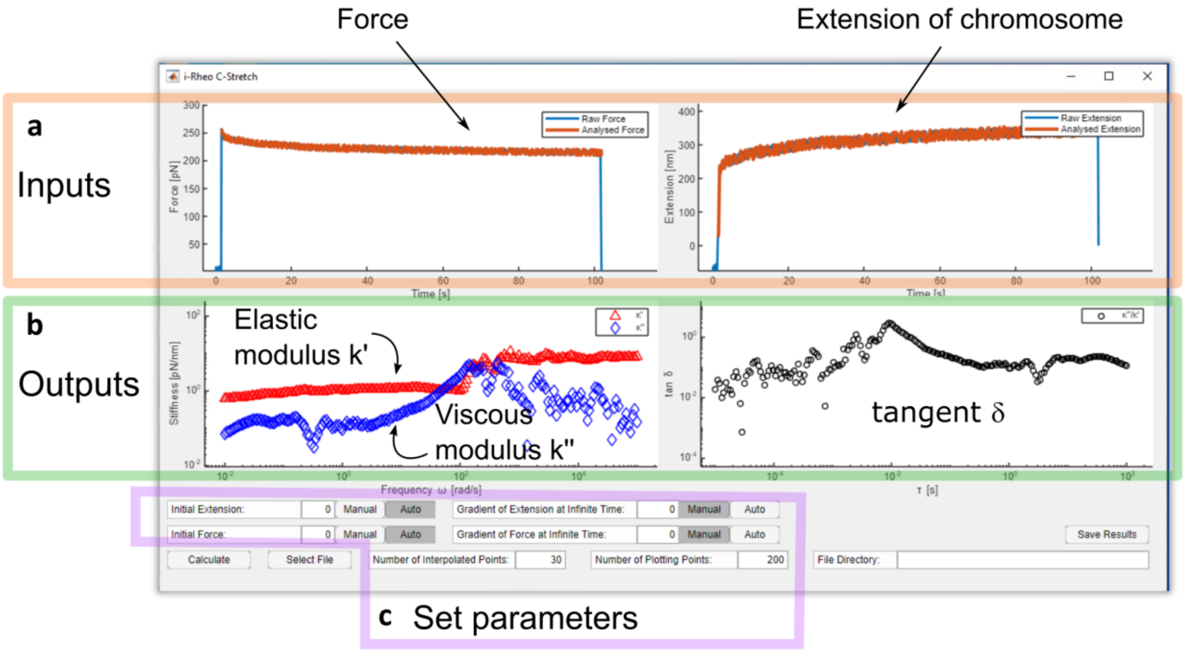

Fig. S5. Screenshot of the i-Rheo C-Stretch app. This plug and play app accepts 3xn datasets of time, force (average between both beads) and relative extension of the chromosome captured at high frequency. a. The inputs are displayed in the two panels at the top and b. the output complex stiffness and $tan\delta$ results are displayed in the bottom two panels of the app. c. Users can toggle between auto detection of parameters (initial and gradient values for force and extension) or manual setting (if known) along with adjusting the number of interpolation points and plotting density. Complex stiffness values can be exported from the app. A detailed guide is available to download with the app (see code availability statement).
