# Supplementary material for: The mitotic chromosome periphery modulates chromosome mechanics
Source: Nat Commun. 2025 Jul 10;16:6399. doi: 10.1038/s41467-025-61755-5 (PMC12246412; doi:10.1038/s41467-025-61755-5)
Supplement: Supplementary file 2 — Reporting Summary [file 41467_2025_61755_MOESM2_ESM.pdf]

nature portfolio

Corresponding author(s): Dr Daniel G. Booth, Prof Amanda J. Wright, Dr Manlio Tassieri, Dr Tania Mendonca

Last updated by author(s): Jun 10, 2025

Reporting Summary

Nature Portfolio wishes to improve the reproducibility of the work that we publish. This form provides structure for consistency and transparency in reporting. For further information on Nature Portfolio policies, see our Editorial Policies and the Editorial Policy Checklist.

Statistics

For all statistical analyses, confirm that the following items are present in the figure legend, table legend, main text, or Methods section.

n/a	Confirmed	
☐	☒	The exact sample size (n) for each experimental group/condition, given as a discrete number and unit of measurement
☐	☒	A statement on whether measurements were taken from distinct samples or whether the same sample was measured repeatedly
☐	☒	The statistical test(s) used AND whether they are one- or two-sided<br>*Only common tests should be described solely by name; describe more complex techniques in the Methods section.*
☒	☐	A description of all covariates tested
☒	☐	A description of any assumptions or corrections, such as tests of normality and adjustment for multiple comparisons
☐	☒	A full description of the statistical parameters including central tendency (e.g. means) or other basic estimates (e.g. regression coefficient) AND variation (e.g. standard deviation) or associated estimates of uncertainty (e.g. confidence intervals)
☐	☒	For null hypothesis testing, the test statistic (e.g. F, t, r) with confidence intervals, effect sizes, degrees of freedom and P value noted<br>*Give P values as exact values whenever suitable.*
☒	☐	For Bayesian analysis, information on the choice of priors and Markov chain Monte Carlo settings
☒	☐	For hierarchical and complex designs, identification of the appropriate level for tests and full reporting of outcomes
☒	☐	Estimates of effect sizes (e.g. Cohen's d, Pearson's r), indicating how they were calculated

Our web collection on statistics for biologists contains articles on many of the points above.

Software and code

Policy information about availability of computer code

Data collection	Bluelake version 2.5.1 (LUMICKS) software with Force Spectroscopy and Force Distance modules were used for optical tweezer manipulations
Data analysis	Custom MATLAB 2022b (MathWorks) scripts were used. The version of scripts (i-Rheo C-Stretch V1.2) used to generate data in this paper can be accessed on Zenodo: https://doi.org/10.5281/zenodo.14527725. Image analysis was performed in Image J version 1.54

For manuscripts utilizing custom algorithms or software that are central to the research but not yet described in published literature, software must be made available to editors and reviewers. We strongly encourage code deposition in a community repository (e.g. GitHub). See the Nature Portfolio guidelines for submitting code & software for further information.

Data

Policy information about availability of data

All manuscripts must include a data availability statement. This statement should provide the following information, where applicable:

- Accession codes, unique identifiers, or web links for publicly available datasets
- A description of any restrictions on data availability
- For clinical datasets or third party data, please ensure that the statement adheres to our policy

Data are available from Zenodo: 10.5281/zenodo.15632578. Data for figures are attached in a Source Data file.

Research involving human participants, their data, or biological material

Policy information about studies with human participants or human data. See also policy information about sex, gender (identity/presentation), and sexual orientation and race, ethnicity and racism.

Reporting on sex and gender	N/A
Reporting on race, ethnicity, or other socially relevant groupings	N/A
Population characteristics	N/A
Recruitment	N/A
Ethics oversight	N/A

Note that full information on the approval of the study protocol must also be provided in the manuscript.

Field-specific reporting

Please select the one below that is the best fit for your research. If you are not sure, read the appropriate sections before making your selection.

☒ Life sciences ☐ Behavioural & social sciences ☐ Ecological, evolutionary & environmental sciences

For a reference copy of the document with all sections, see nature.com/documents/nr-reporting-summary-flat.pdf

Life sciences study design

All studies must disclose on these points even when the disclosure is negative.

Sample size	Sample sizes have been included in Figure legends where data are displayed. No statistical method was used to predetermine sample size but sample sizes are typical for similar micromechanical studies.
Data exclusions	Only chromosomes between 2-5 µm in length were analysed to ensure single chromosomes with telomeric attachment were being tested. Damaged and unravelled chromosomes were discarded on visual inspection. This has been stated clearly in the Methods section. Data were also excluded if chromosomes detached from the bead handles during the optical tweezer manipulations.
Replication	All attempts are replication were successful. Data were collected over several months and two different instruments (at University of Nottingham and University of Kent) showing consistent results. Each experiment type had at least three independent replicates (from tissue culture to optical tweezer manipulation).
Randomization	Experiments were not randomised but data collection methods were kept consistent and scripts were used to automate analysis to remove bias. Order of data collection for each treatment was swapped between repeats to avoid any bias.
Blinding	No blinding was applied to data collection or analysis.

Reporting for specific materials, systems and methods

We require information from authors about some types of materials, experimental systems and methods used in many studies. Here, indicate whether each material, system or method listed is relevant to your study. If you are not sure if a list item applies to your research, read the appropriate section before selecting a response.

Materials & experimental systems

n/a	Involved in the study	
☐	☒	Antibodies
☐	☒	Eukaryotic cell lines
☒	☐	Palaeontology and archaeology
☒	☐	Animals and other organisms
☒	☐	Clinical data
☒	☐	Dual use research of concern
☒	☐	Plants

Methods

n/a	Involved in the study	
☒	☐	ChIP-seq
☒	☐	Flow cytometry
☒	☐	MRI-based neuroimaging

Antibodies

Antibodies used	Primary antibodies were used as follows: NPM1 (Mouse monoclonal, #32-5200 (Thermo fisher, Waltham, Massachusetts, USA) 1:60; Nucleolin (Rabbit polyclonal, Abcam, Cambridge, UK, #ab22758) at dilution of 1:100.<br>Fluorescence-labelled secondary antibodies: anti-mouse IgG-TRITC (Jackson ImmunoResearch, Ely, UK) and anti-Rabbit IgG-TRITC (Jackson ImmunoResearch, Ely, UK) were applied at dilution of 1:400.
Validation	NPM1 (Mouse monoclonal, #32-5200 (Thermo fisher, Waltham, Massachusetts, USA) was validated by ThermoFisher using Knockdown to ensure that the antibody binds to the antigen stated. Nucleolin (Rabbit polyclonal, Abcam, Cambridge, UK, #ab22758) was validated using ICC/IF, IHC-Fr, IHC-P and IP by Abcam. For details see supplier websites.

Eukaryotic cell lines

Policy information about cell lines and Sex and Gender in Research

Cell line source(s)	HeLa parental cell line was sourced from DSMZ
Authentication	DSMZ authenticated the cell line using short tandem repeat (STR) DNA typing
Mycoplasma contamination	Monthly mycoplasma testing was performed using PlasmoTest mycoplasma contamination detection kit (InvivoGen, Toulouse, France) and all cell lines used were confirmed to be negative for mycoplasma contamination.
Commonly misidentified lines<br>(See ICLAC register)	No commonly misidentified cell lines were used in the study

Plants

Seed stocks	N/A
Novel plant genotypes	N/A
Authentication	N/A
